# NRF2 and STAT3: friends or foes in carcinogenesis?

**DOI:** 10.1007/s12672-023-00644-z

**Published:** 2023-03-31

**Authors:** Andrea Arena, Maria Anele Romeo, Rossella Benedetti, Maria Saveria Gilardini Montani, Roberta Santarelli, Roberta Gonnella, Gabriella D’Orazi, Mara Cirone

**Affiliations:** 1 Department of Experimental Medicine, Sapienza University of Rome, Viale Regina Elena 324, 00161 Rome, Italy; 2 Department of Neurosciences, Imaging and Clinical Sciences, University “G. D’Annunzio”, 66013 Chieti, Italy; 3 School of Medicine, UniCamillus International University, 00131 Rome, Italy

**Keywords:** NRF2, STAT3, p62/SQSTM1, Cytokines, DDR

## Abstract

NRF2 is a transcription factor that plays a pivotal role in carcinogenesis, also through the interaction with several pro-survival pathways. NRF2 controls the transcription of detoxification enzymes and a variety of other molecules impinging in several key biological processes. This perspective will focus on the complex interplay of NRF2 with STAT3, another transcription factor often aberrantly activated in cancer and driving tumorigenesis as well as immune suppression. Both NRF2 and STAT3 can be regulated by ER stress/UPR activation and their cross-talk influences and is influenced by autophagy and cytokines, contributing to shape the microenvironment, and both control the execution of DDR, also by regulating the expression of HSPs. Given the importance of these transcription factors, more investigations aimed at better elucidating the outcome of their networking could help to discover new and more efficacious strategies to fight cancer.

## NRF2

Nuclear factor E2-related factor 2 (NRF2) is a transcription factor that plays a pivotal role in cytoprotection, mainly because it induces the transcription of phase I and II phase I detoxification enzymes, limiting oxidative stress [1]. NRF2 activity is particularly important for the survival of cancer cells that, due to external and internal causes, are characterized by high level of reactive oxygen species (ROS), whose production may be further enhanced by exposure to anti-cancer treatments. Interestingly, NRF2 is considered a double face molecule, as its-activity, that helps to prevent tumor onset, can also sustains growth and survival of established tumor cells [2]. The canonical activation of NRF2 is caused by oxidative stress that induces the detachment of NRF2 from Kelch-like ECH-associated protein 1 (KEAP1), the most important negative regulator of NRF2 [3]. In this conditions, NRF2 free from the holding of KEAP1, can translocate into the nucleus, form heterodimers with one of the small Maf (musculoaponeurotic fibrosarcoma oncogene homolog) proteins and recognize the antioxidant response elements (AREs), the enhancer sequences present in the regulatory regions of NRF2 target genes [4]. Once in the nucleus, NRF2, besides the de-toxifying enzymes, affects the transcription of a variety of molecules, as recently revised by Tonelli et al. [1]. NRF2 can be also stabilized by SQSTM1/p62, protein that promotes KEAP1 degradation [5]. This results in the non-canonical activation of NRF2, which can be the consequence of autophagy reduction, given that SQSTM1/p62 is a protein mainly degraded through autophagy and thus accumulates when such process is impaired [6]. Autophagy, is a self-eating mechanism, representing together with proteasome, the most important catabolic route of the cells. Basally activated in almost all cellular types, autophagy is particularly important in the survival of cancer cells that are forced to live in condition of shortage of oxygen and nutrients [7]. Moreover, the activation of NRF2 is regulated, through a feed-back mechanism, by Batch1, a transcriptional repressor of NRF2 activated by NRF2 itself [8].

## STAT3

Signal transducer and activator of transcription 3 (STAT3) is a multifunctional transcription factor, classically activated by the Interleuchin-6 (IL-6)-type family cytokines, through Janus kinase (JAK) [9]. STAT3 establishes with IL-6 as well as with VEGF and IL-10, a positive feed-back loop, in which these cytokines activate STAT3 and this transcription factor, in turn, sustains their production. STAT3 interacts with several cellular pathways involved in cancer cell proliferation, progression and immune dysfunction. Aberrant activation of STAT3, due to phosphorylation of a tyrosine residue (Y705) and, in some cases of serine residue (S727), occurs in about 70% of cancers, either of solid and hematologic origin. STAT3 is generally considered to be an oncogene, although in some particular contexts, it may play a tumor suppressing role [10, 11]. Interestingly, the inhibition of STAT3 in cancer may give the double advantage to impair cell survival and concomitantly reactivate the anti-cancer immune response [12]. Of note, unphosphorylated STAT3 can also mediate the transcriptional activation of some genes, such as for example those encoding for STAT3 itself or those involved in the control of cell cycle progression [13].

Besides cytokines, STAT3 can be activated also by tyrosine kinases, such as v-Fps, v-Ros, Etk/BMX and v-Abl or by proteins encoded by oncoviruses such as Epstein-Barr virus (EBV) [14].

## NRF2/STAT3 interplay involves p62/SQSTM1 and autophagy

The interplay between NRF2 and STAT3 is quite controversial and interestingly, it may also involve autophagy. Therefore, in this perspective, we will discuss the literature reports and attempt to shed some light on this interplay, particularly in the context of cancer cells.

Autophagy is a process controlled by several molecular pathways, including phosphatidylinositol 3-kinase (PI3K)/AKT/mammalian target of the rapamycin (mTOR) (PI3K/AKT/mTOR) [15] and STAT3 [16], whose impact on autophagy depends not only on the status of their phosphorylation, but also on their subcellular localization. Indeed, it has been reported that STAT3 cytoplasmic localization inhibits PKR activity and autophagy in osteosarcoma cells [17]. Moreover, in its unphosphorylated state, STAT3 may promote heterochromatin formation in lung cancer cells, suppressing cell proliferation [18]. Interestingly, autophagy may be dysregulated by external insults, for example microbial infection, either mediated by bacteria [19] or viruses [20], including the oncoviruses EBV, Kaposi’s sarcoma-associated herpesvirus (KSHV) and Hepatitis C Virus (HCV), as we have previously reported [21–24]. PI3K/AKT/mTOR and STAT3 are both considered to be mainly negative regulators of autophagy, as they can mediate an inhibitory effect on one or more of the different phases of the autophagic process. In this regard, we have previously observed that EBV infection of primary B lymphocytes [23] or KSHV infection of HUVEC cells [24] activate STAT3 and mTOR, respectively, to inhibit autophagy. As B lymphocytes and HUVEC are cells from which gammaherpesvirus-associated cancers arise, the inhibition of autophagy represents an important goal achieved by these viruses. Indeed, autophagy has been reported to put a brake on oncogenic transformation [25]. Moreover, it must be considered that autophagy promotes viral elimination through xenophagy [26], contributes to antigen presentation to dendritic cells (DCs) and to macrophages formation [27]. As it plays a key role in anti-viral immune response, autophagy inhibition by viruses may represent a defense mechanism. Moreover, they can also usurp the autophagic machinery for their own purpose, e.g. to facilitate the intracellular transportation of viral particles [28, 29].

**Fig. 1:**
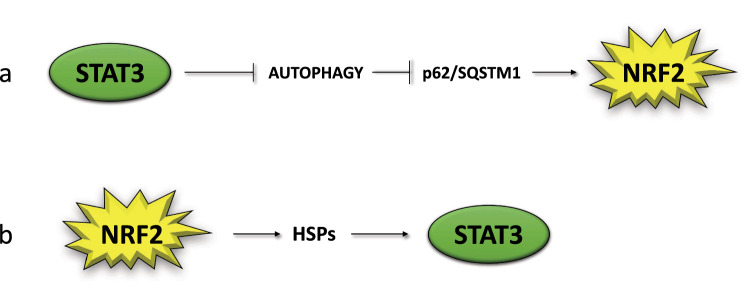
Scheme illustrating the reciprocal positive regulation of NRF2 and STAT3

**Fig. 2:**
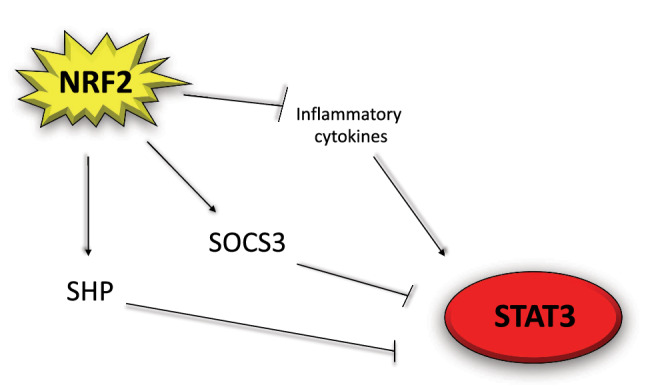
Scheme illustrating the mechanisms through which NRF2 can inhibit STAT3

Among other consequences, the impairment of autophagy leads to SQSTM1/p62 accumulation [6], which promotes NRF2 stabilization and activation, upregulating the anti-oxidant response [30]. This represents a compensatory mechanism that allows restrain the increase of ROS occurring in autophagy-deficient cells, as it occurs for example in the case of EBV-infection of primary monocytes. In these cells, the increase of p62/SQSTM1, by leading to a reduction of ROS, impairs the in vitro differentiation of monocytes into dendritic cells (DCs) or macrophages [27]. p62/SQSTM1/NRF2 axis activation may thus represent a strategy exploited by EBV to inhibit the formation of cells, such as DCs, that play a key role in initiating an immune response towards a new antigen [31, 32]. From these evidences, it emerges that autophagy and p62/SQSTM1 may represent a link between mTOR and STAT3 activation and NRF2. Previous studies have started to explore some aspects of the relationship between NRF2 and mTOR [33], while in this perspective we will mainly focus on STAT3/NRF2 interplay. If, as said above, STAT3 may activate NRF2, through a STAT3/p62SQSTM1 axis (Fig. 1a), NRF2 may play an opposite effect, as it has been reported that AMPK-driven STAT3 inactivation involves Nrf2-small heterodimer protein (SHP) signaling cascade [34] (Fig. 2). Furthermore, among the components of the NRF2 interactome there is PPARγ, as NRF2 binds to its promoter, stimulating its transcription and engages with it a mutual feedback regulation [35]. Interestingly, PPARγ has been shown to induce STAT3 inactivation in pancreatic acinar cells [36].

## Role of NRF2 and STAT3 in inflammation

Among the numerous processes controlled by NRF2, there is the inflammatory process [37], that, when lasts for long time, can sustain all steps of carcinogenesis [38]. NRF2 activation may reduce inflammation, as another compensatory mechanism through which cells attempt to defend themselves from oncogenic transformation, when autophagy is dysfunctional. Accordingly, we have found that the activation of NRF2 in primary B lymphocytes, in which the autophagic process is inhibited by EBV infection, restrains IL-6 cytokine production, besides lowering intracellular ROS [39], and both these effects counteract the in-vitro immortalization of B cells driven by the virus. The role of NRF2 in tumor prevention in this setting was supported by the experiments of silencing, pharmacological inhibition by Brusatol and on the other hand, by experiments in which NRF2 was activated by Sulforaphane. However, NRF2, depending on the initial steps of oncogenic transformation or in already transformed cells, can play opposite effects in carcinogenesis [2]. This difference mainly relies on the capacity of NRF2 to reduce ROS, which are the molecules most responsible for DNA damage, mutations and promotion of tumorigenesis, but are also those that can impair the survival of cancer cells, when their level become too high. It has been reported that the treatment by Dimethyl fumarate (DMF), an activator of NRF2, repressed JAK1 and STAT3 phosphorylation in hepatocellular carcinoma [40]. Accordingly, in a recent study, we have shown that NRF2 activation by DMF reduces ROS, IL-6 and IL-10 production by B lymphoma cells, resulting, also in this case, in a strong de-phosphorylation of STAT3 (Fig. 2) and in an impairment of cell survival [41]. These results suggest that it is not NRF2 that behaves differently during cancer formation or cancer survival/progression but are rather the effects that it produces that may play opposite roles in the different phases of tumorigenesis.

Interestingly, a recent study has reported that Brusatol can also lead to the inhibition of STAT3 and of that of the upstream kinases responsible for its activation, in Head and Neck Squamous Cell Carcinoma [42], underscoring the complexity of NRF2 and STAT3 interplay in cancer and thus the difficulty to develop new drugs targeting these pathways [43]. Moreover, it should be considered that the specificity of Brusatol, commonly used to inhibit NRF2, remains to be better elucidated [44]. Of note, differently from NRF2, the activation of STAT3 can promote cell survival/proliferation, either during the first steps of carcinogenesis [23] and in established cancer cells [45–47], also because its activation may be sustained by NF-κB, as in the case of glioblastoma [48]. Another reason for such difference may be the fact that STAT3 promotes, rather than reducing, the production of pro-inflammatory and pro-angiogenetic cytokines such as IL-6 and VEGF, establishing with these cytokines a positive feed-back loop [49]. However, there are some cases in which STAT3 may play an anti-survival role, as reported, for example, in studies performed on mammary gland [50]. Moreover, among the other targets, STAT3 can also upregulate the expression of IL-6 inhibitory molecule Suppressor of Cytokine Signaling 3 (SOCS3) [51] and interestingly NRF2 has been reported to enhance SOCS3-dependent feedback inhibition on JAK2/STAT3, in hepatic stellate cells (Fig. 2) [52]. To recapitulate this complex landscape, it seems that the unbalance of NRF2 activation, either towards its hyperactivation or toward its inhibition, may affect STAT3 activation, cytokines release and the survival of cancer cells. Of note, cytokines may function not only as growth factors for several cancer cells that are responsible for their production, but may also strongly shape of the tumor microenvironment [53] and unbalance the pro-tumorigenic or anti-tumorigenic activity of immune system cells. For example, the release of cytokines such as IL-23, which may be regulated by STAT3 or by STAT3/NRF2 interplay can promote tumor growth [54] or contribute to immune dysfunction of dendritic cells, following KSHV infection [55]. Last but not least, cytokines may affect the activity of stromal cells such as fibroblasts and endothelial cells that may contribute to their production [56], and influence the progress of cancer [57].

## NRF2 and STAT3 are influenced by post-translational modifications

Post-translational modifications, such as phosphorylation, can strongly influence the activity of NRF2. Phosphorylation of NRF2 may occur at different residues and may be mediated by several kinases, resulting either in the activation or inhibition of NRF2 [58]. Among the kinases able to phosphorylate NRF2 there is Protein kinase R (PKR)-like endoplasmic reticulum kinase (PERK) [59], one of the three Unfolded Protein Response (UPR) sensors, which can be activated in response to Endoplasmic Reticulum (ER) stress. Therefore, besides autophagy, another adaptive response, namely UPR, impinges on the activation of NRF2. Of note, PERK can phosphorylate and activate also STAT3 [60]. Thus, it emerges that in stressed cells, the phosphorylation of both NRF2 and STAT3 may occur and usually contribute to cell survival. However, it is important to consider that NRF2 activation can be also inhibited in the course of UPR, through the activation of Glycogen synthase kinase 3 beta (GSK3beta) [58], highlighting once again the complexity of NRF2 regulation, also in stressed cells.

Among other post-translational modifications, methylation has been reported to influence NRF2 and STAT3 activity, as indeed methylation has been found to modify several nonhistone proteins including NRF2 and STAT3. Regarding NRF2, it has been shown that methylation can occur at arginine 437 residue and that this effect can moderately affect NRF2 activity [61, 62]. Regarding STAT3, it has been reported that it can be di- or trimethylated on K140 or K180 by the histone methyltransferase SET9 (SET domain containing lysine methyltransferase 9) or EZH2 (enhancer of zeste homolog 2), respectively, and that such post-translational modifications may affect STAT3-mediated transcription [63–65]. Interestingly, STAT3 can in turn regulates methylation of several promoters, as for example it promotes SOCS3 hypermethylation by increasing DNMT1 activity [66] and intriguingly, the acetylation of STAT3 may regulate its-mediated methylation [67]. These studies suggest the post-translational modifications of NRF2 and STAT3 are complex and context-specific, and thus deep investigations are required to clarify them.

## NRF2 and STAT3 gene transcription may be regulated by methylation

Of note, methylation of NRF2 and STAT3 promoters can also influence the expression and activity of these transcription factors. For example, in the context of Alzheimer Disease, NRF2 activity is reduced by methylation and the DNA methyltransferases (Dnmts) inhibitor 5-aza-2′-deoxycytidine (5-Aza) may enhance NRF2 expression and transcriptional activity [68]. Regarding NRF2 methylation in cancer, it has been reported that no significant association could be found between NRF2 promoter demethylation and the clinicopathological features of colon cancer patient samples [69]. Another study has reported that sulforaphane, that promotes the demethylation of NRF2 promoter region, increases the activation of NRF2 in CaCo2 cells [70]. Regarding STAT3, it has been reported that 5-AZA may reduce its activation in acute myeloid leukemia cells by re-expression of silenced SHP-1, a negative regulator of the JAK/STAT pathway [71].

## NRF2/STAT3 interplay affects DDR

An interesting point that deserves to be discussed is how the interplay between NRF2 and STAT3 affects the DNA damage response (DDR). In the physiological execution of DDR, Heat Shock Proteins (HSPs) plays a pivotal role, as many molecules, involved in both single- and double-stranded DNA breaks, are HSP clients [72]. For example, small HSPs, such as HSP27 can stabilize and prevent the proteasomal degradation of the protein kinase Ataxia telangiectasia mutated (ATM) while HSP90 contributes to the correct folding of proteins involved in DNA Homologous Repair (HR) such as RAD51 [72]. Interestingly, HSPs localized into the nucleus also contribute to cell protection from oxidative stress [73]. NRF2, together with Heat shock factor 1 (HSF1), represents the main transcription factor regulating the expression of HSP [74] and therefore NRF2 can indirectly control the DNA damage repairing pathways. To recapitulate, NRF2 activation can be induced by STAT3/SQSTM1/p62 axis and lead to the upregulation of the HSPs and thus sustain DDR. Moreover, NRF2 has been reported to directly regulate the transcription of DDR molecules such as ATM and Ataxia telangiectasia and Rad3-related protein (ATR), kinases that sense DNA damage and activate the repairing signaling cascade [75]. It is important to point out that DDR plays a key role not only in tumor prevention but also in chemoresistance of established cancer, unveiling how another process controlled by NRF2 can induce both desirable and undesirable effects, depending if it occurs in the initial or in the advanced phases of carcinogenesis. We have observed that NFR2 silencing in B lymphocytes undergoing EBV-driven transformation reduced the expression of ATM [39]. Ongoing studies in our laboratory suggest that NRF2 may contribute to sustain the expression of ATM also in B cell lymphoma cells (unpublished data). It seems that, besides through a direct transcriptional control, ATM expression may be regulated by NRF2 through the transcription of HSPs such as HSP27, of which, as said above, ATM may be a client protein [72].

HSPs could contribute to STAT3 activation mediated by NRF2, reported in previous studies [42] as, among many other molecules, HSPs may stabilize kinases such as JAK2, involved in STAT3 phosphorylation (Fig. 1b) [76]. In the interplay between NRF2 and STAT3, may play a role p53, as this onco-suppressor, activated by STAT3 inhibition [77, 78] as well as by NRF2 inhibition [79] is able, in turn, to inhibit both STAT3 [80] and NRF2 [81]. In cancer cells carrying mutp53, the mutant proteins could also act as a bridge between NRF2 and STAT3, although differently from wtp53, mutp53 may activate both transcription factors [82–84].

## Conclusions

**Fig. 3:**
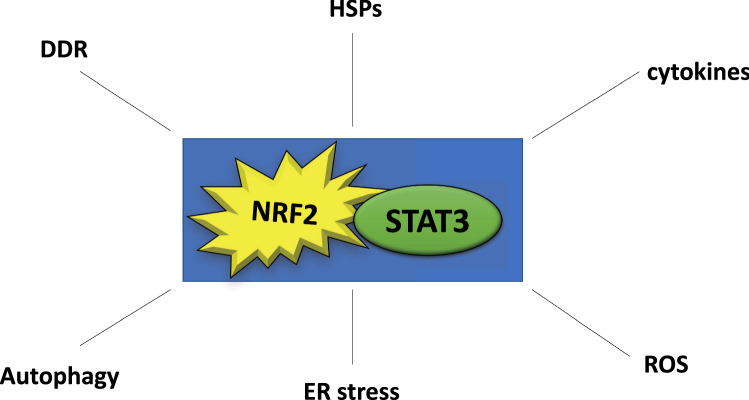
Scheme illustrating how NRF2/STAT3 interplay affect multiple processes and molecules

In conclusion, with this perspective, we attempted to have shed some light into the complex interplay between NRF2 and STAT3, pathways playing a key role in cancers, either of hematologic and solid origin. As these transcription factors may either activate or inhibit each other (Figs. 1 and 2) and may play opposite roles in cancer prevention while acting similarly in established cancers, their interplay is very intriguing and is worth of further investigations. A part from the cross-talk between them, NRF2 and STAT3 interplay controls a variety of processes and pathways (Fig. 3) that play a key role in cancer as well as in several other diseases. Moreover, elucidating the articulated network of NRF2, influenced by many unilateral and reciprocal interactions, recapitulated in the “NRF2-ome” [85] or that of STAT3 [86] will likely open new avenues in the prevention or treatment of cancer.

## Data Availability

The data are available upon request to the corresponding author.

## Abbreviations

NRF2: Nuclear factor E2-related factor 2
ROS: Reactive oxygen species
KEAP1: Kelch-like ECH-associated protein 1
AREs: Antioxidant response elements
SQSTM1: Sequestosome 1
STAT3: Signal transducer and activator of transcription 3
IL: Interleukin
JAK: Janus kinase
VEGF: Vascular Endothelial Growth Factor
EBV: Epstein-Barr virus
PI3K: Phosphatidylinositol 3-kinase
mTOR: Mammalian target of the rapamycin
KSHV: Kaposi’s sarcoma-associated herpesvirus
HCV: Hepatitis C Virus
DCs: Dendritic cells
SHP: Small heterodimer protein
SOCS3: Suppressor of Cytokine Signaling 3
PERK: Protein kinase R (PKR)-like endoplasmic reticulum kinase
UPR: Unfolded Protein Response
ER: Endoplasmic Reticulum
GSK3beta: Glycogen synthase kinase 3 beta
Dnmts: DNA methyltransferases
5-Aza: 5-Aza-2′-deoxycytidine
SHP-1: Src homology-2 (SH2)-containing protein-tyrosine phosphatase
DDR: DNA damage response
HSPs: Heat Shock Proteins
ATM: Ataxia telangiectasia mutated
HR: Homologous Repair
HSF1: Heat shock factor 1
ATR: Ataxia telangiectasia and Rad3-related protein
